# Genetically reprogrammed BMSCs with Lamin B1 depletion enhance bone regeneration for osteoporosis therapy

**DOI:** 10.3389/fbioe.2026.1803514

**Published:** 2026-04-01

**Authors:** Xuekun Fu, Shaochuan Huo, Chunhao Cao, Hanson Hsu, Jie Huang, Yuhe Lei, Jie Li, Peng Liu, Jincheng Zeng, Songqing Lin, Chao Liang

**Affiliations:** 1 Shenzhen Hospital (Futian) of Guangzhou University of Chinese Medicine, Shenzhen, China; 2 Department of Systems Biology, School of Life Sciences, Southern University of Science and Technology, Shenzhen, China; 3 Laboratory of Gene Regulation and Signal Transduction, Departments of Pharmacology and Pathology, School of Medicine, University of California, San Diego, La Jolla, CA, United States; 4 Department of Laboratory Medicine, Peking University Shenzhen Hospital, Shenzhen, China; 5 Dongguan Key Laboratory of Medical Bioactive Molecular Developmental and Translational Research, Guangdong Provincial Key Laboratory of Medical Immunology and Molecular Diagnostics, Guangdong Medical University, Dongguan, China; 6 Shenzhen Futian District Maternal and Child Health Hospital, Shenzhen, China; 7 Institute of Integrated Bioinfomedicine and Translational Science (IBTS), School of Chinese Medicine, Hong Kong Baptist University, Hong Kong SAR, China; 8 State Key Laboratory of Proteomics, National Center for Protein Sciences (Beijing), Beijing Institute of Lifeomics, Beijing, China

**Keywords:** BMSCs, KAT2A/GCN5, Lamin B1, osteoporois, regeneration

## Abstract

**Background:**

Mesenchymal stem cell (MSC) dysfunction contributes to impaired bone regeneration in osteoporosis. Lamin B1, a nuclear lamina protein implicated in stem cell aging, has an unclear role in osteogenesis.

**Methods:**

We examined Lamin B1 expression during osteogenic differentiation, assessed its pathological alterations in OVX-derived BMSCs, and generated Lamin B1–knockout MSCs to evaluate effects on osteogenesis, migration, and KAT2A regulation. We performed transcriptomic profiling and intra–bone marrow transplantation in OVX mice to determine functional relevance.

**Results:**

Lamin B1 expression progressively decreased during osteogenic induction but was markedly upregulated in OVX BMSCs, where it correlated with impaired migration. Lamin B1 deletion enhanced alkaline phosphatase activity, mineralization, and migration. Mechanistically, Lamin B1 interacted with KAT2A and promoted its ubiquitin-dependent degradation, thereby reducing KAT2A protein stability. Knockdown-induced transcriptional changes indicated activation of osteogenic and migration-related pathways. *In vivo*, Lamin B1-deficient MSCs showed improved engraftment and substantially enhanced bone regeneration, reflected by increased BMD, BV/TV, MAR, and BFR in OVX mice.

**Conclusion:**

Lamin B1 depletion enhances BMSC osteogenesis by preventing KAT2A degradation. Lamin B1-deficient BMSCs provide a promising gene-enhanced cell therapy strategy for osteoporosis.

## Introduction

Bone homeostasis depends on the coordinated balance between osteoblast-mediated bone formation and osteoclast-mediated bone resorption (Wang et al., 2022; Castellani et al., 2025). Disruption of this balance results in progressive bone loss and ultimately leads to osteoporosis, a chronic skeletal disorder characterized by reduced bone mass, deterioration of trabecular architecture, and heightened fracture susceptibility (Tuckermann and Adams, 2021; Wang et al., 2022). With the global population aging, osteoporosis imposes a substantial clinical and economic burden (Chandra and Rajawat, 2021). Current antiresorptive and anabolic therapies can slow bone loss, yet their long-term effectiveness is limited, and they do not directly address the intrinsic cellular defects that impair bone formation (Noh et al., 2020). These limitations highlight the importance of defining the molecular mechanisms that regulate osteoblast development and identifying strategies capable of restoring endogenous regenerative capacity.

Mesenchymal stem cells (MSCs), particularly bone marrow–derived MSCs (BMSCs), serve as the primary progenitors of osteoblasts and therefore play a central role in skeletal maintenance (Deschaseaux et al., 2010; Wang et al., 2013). During aging and osteoporosis, BMSCs show diminished proliferation, impaired osteogenic differentiation, and a shift toward adipogenic commitment, all of which contribute to reduced bone formation (Kim et al., 2012; Infante and Rodríguez, 2018). The lineage commitment of MSCs is controlled by an intricate interplay of transcriptional, mechanical, and epigenetic cues (Wu et al., 2024). Established regulators of osteogenesis include Runx2, Osterix, Wnt/β-catenin, and BMP signaling (Zhu et al., 2024). Parallel to these pathways, epigenetic mechanisms—especially histone acetylation and other chromatin modifications—strongly influence osteogenic gene activation (Wang X. et al., 2023; Wang Z. et al., 2023). Histone acetyltransferases such as KAT2A promote transcription of osteogenic programs (Jing et al., 2018), yet the upstream structural factors that modulate the stability and activity of these chromatin regulators remain poorly understood. Given that nuclear architecture integrates mechanical and epigenetic information, clarifying how it contributes to MSC fate determination may reveal new strategies for restoring osteogenic capacity in bone-loss disorders.

The nuclear lamina, composed of type A lamins (lamins A/C) and type B lamins (Lamin B1/B2), forms a filamentous scaffold beneath the inner nuclear membrane and contributes to nuclear mechanics, chromatin organization, transcriptional regulation, and mechanotransduction (Shimi et al., 2008; Vahabikashi et al., 2022). Increasing evidence suggests that lamins influence stem cell differentiation by altering nuclear tension and modulating chromatin accessibility (Heo et al., 2018; Bitman-Lotan and Orian, 2021). Lamin A/C deficiency disrupts osteogenesis and skeletal integrity, underscoring the importance of nuclear structure in bone biology (Rauner et al., 2009). By contrast, the role of Lamin B1 in MSC fate remains largely undefined. Although Lamin B1 regulates cellular senescence, genome stability, and lineage transitions in other tissues (Gigante et al., 2017; Chen et al., 2019; Bin Imtiaz et al., 2021), its contribution to osteogenic regulation and osteoporosis pathogenesis is unknown. This knowledge gap is particularly significant because MSCs are highly sensitive to nuclear mechanics and epigenetic regulation, processes that are tightly coupled to the nuclear lamina.

MSC-based therapies have emerged as a promising approach for osteoporosis and other degenerative skeletal diseases (Chen et al., 2021; He et al., 2021; Jiang et al., 2021; Souza et al., 2024). However, the clinical efficacy of MSC-based therapy for osteoporosis remains limited due to reduced engraftment efficiency and functional impairment of MSCs under osteoporotic conditions, highlighting the need for strategies that enhance intrinsic stem cell function. Genetic enhancement offers a potential means to overcome these limitations. Given the central role of the nuclear lamina in chromatin regulation and stem cell function, modulating Lamin B1 expression may reprogram MSCs toward a pro-osteogenic state. In this work we examined the role of Lamin B1 in MSC osteogenic differentiation and bone regeneration. We characterized its expression dynamics, evaluated its functional relevance using CRISPR/Cas9-mediated knockout models, investigated the mechanism with a focus on KAT2A stability, and assessed the therapeutic efficacy of Lamin B1-deficient MSCs in an ovariectomy-induced osteoporosis model. Our findings reveal Lamin B1 as a previously unrecognized negative regulator of MSC osteogenesis and support its potential as a target for gene-enhanced stem cell therapy in osteoporosis.

## Results

### Lamin B1 expression decreased during osteogenic differentiation of mesenchymal stem cells

**FIGURE 1:**
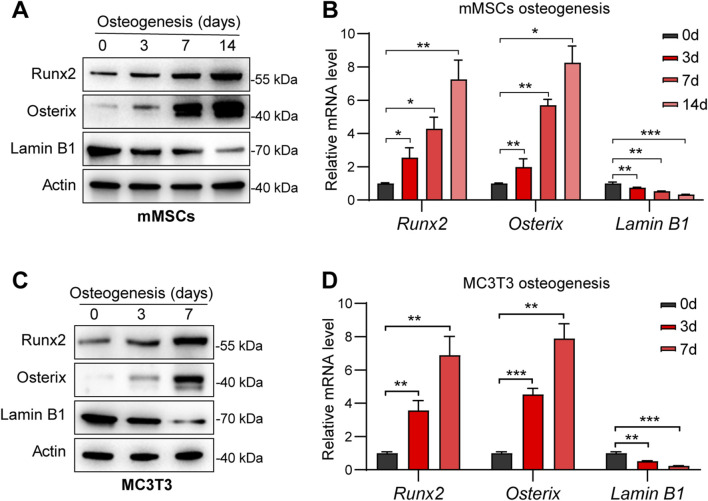
Lamin B1 expression progressively decreases during osteogenic differentiation of mMSCs and MC3T3 cells. **(A)** Representative Western blot analysis showing the expression profiles of Runx2, Osterix, Lamin B1, and Actin in mMSCs undergoing osteogenic induction over 14 days. **(B)** Quantitative analysis of *Runx2*, *Osterix*, and *Lamin B1* mRNA levels in mMSCs at days 0, 3, 7, and 14 of osteogenic differentiation, normalized to β-actin. Data are presented as mean ± SD (n = 3). *P < 0.05, **P < 0.01, ***P < 0.001 versus day 0. **(C)** Representative Western blot analysis of Runx2, Osterix, Lamin B1, and β-actin expression in MC3T3 cells during osteogenic induction over 7 days. **(D)** Quantitative analysis of *Runx2*, *Osterix*, and *Lamin B1* mRNA levels in MC3T3 cells at days 0, 3, and 7, normalized to β-actin. Data are presented as mean ± SD (n = 3). **P < 0.01, ***P < 0.001 versus day 0.

To determine the temporal expression pattern of Lamin B1 during osteogenic differentiation, we first induced osteogenesis in mouse mesenchymal stem cells (mMSCs) and MC3T3 pre-osteoblasts. In mMSCs, Western blot analysis revealed progressive increases in the osteogenic markers Runx2 and Osterix throughout the 14 days induction period, confirming successful differentiation (Figure 1A). In contrast, Lamin B1 protein levels steadily declined as osteogenesis proceeded. Quantitative RT-PCR analysis showed a similar pattern at the mRNA level, with significant downregulation of *Lamin B1* beginning on day 7 and becoming more pronounced by day 14 (Figure 1B). We next assessed whether this pattern was conserved in pre-osteoblast MC3T3 cells. Consistent with mMSC findings, MC3T3 cells exhibited upregulation of Runx2 and Osterix during osteogenic differentiation, whereas Lamin B1 expression decreased sharply over 7 days (Figure 1C). mRNA quantification confirmed significant suppression of *Lamin B1* during differentiation (Figure 1D). Together, these data indicate that Lamin B1 is inversely correlated with osteogenic progression, suggesting a potential inhibitory role of Lamin B1 in osteoblast lineage commitment.

### Lamin B1 was upregulated in BMSCs from OVX mice and correlates with impaired migration capacity

**FIGURE 2:**
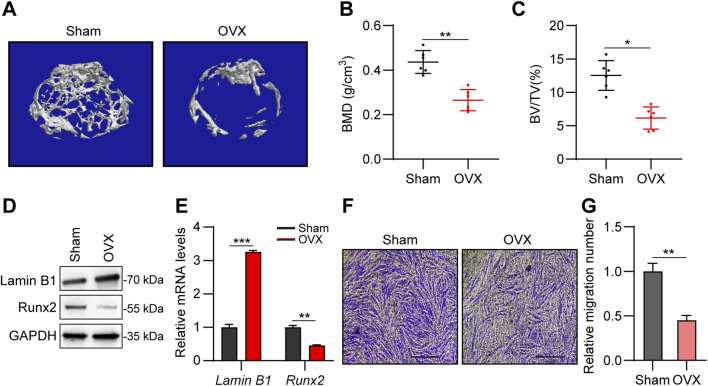
Ovariectomy alters bone microarchitecture and modulates Lamin B1 expression and migratory capacity of BMSCs. **(A)** Representative micro-CT images of the proximal femur from sham-operated and OVX mice. **(B)** Quantification of bone mineral density (BMD) in the femoral neck region (n = 6). **(C)** Quantification of bone volume to total volume ratio (BV/TV) in the femoral neck region (n = 6). **(D)** Western blot analysis of Lamin B1 and Runx2 protein expression in BMSCs isolated from sham-operated and OVX mice, with GAPDH as a loading control. **(E)** Quantitative analysis of *Lamin B1* and *Runx2* mRNA levels in BMSCs, normalized to GAPDH. **(F)** Representative images from the Transwell migration assay performed on BMSCs derived from sham-operated and OVX mice. **(G)** Quantification of migrated cells expressed as a percentage of total cells. Data are presented as means ± SD, *P < 0.05, **P < 0.01 and ***P < 0.001.

Given the relevance of MSC dysfunction in osteoporosis, we examined Lamin B1 expression in BMSCs isolated from ovariectomized (OVX) mice. Micro-CT analysis confirmed typical osteoporotic bone phenotypes, including elevated trabecular porosity and reduced bone mass in OVX animals compared with sham controls (Figure 2A). Quantitative measurements of BMD and BV/TV revealed significant reductions in OVX mice (Figures 2B,C). Western blot analyses showed that Lamin B1 protein expression was markedly elevated in BMSCs from OVX mice, whereas the osteogenic transcription factor Runx2 was reduced (Figure 2D). These expression patterns were consistent at the mRNA level, where *Lamin B1* transcripts were significantly increased and *Runx2* transcripts significantly decreased (Figure 2E). Functionally, BMSCs derived from OVX mice exhibited markedly impaired migration. Transwell assays revealed fewer migrating OVX BMSCs compared with sham controls (Figure 2F), and quantification confirmed a significant reduction in migratory cell numbers (Figure 2G). These findings suggest that pathological upregulation of Lamin B1 in osteoporotic BMSCs is associated with impaired osteogenic potential and deficient migration, consistent with a detrimental role for Lamin B1 in MSC function.

### Depletion of Lamin B1 enhanced osteogenic differentiation and migration of primary BMSCs

**FIGURE 3:**
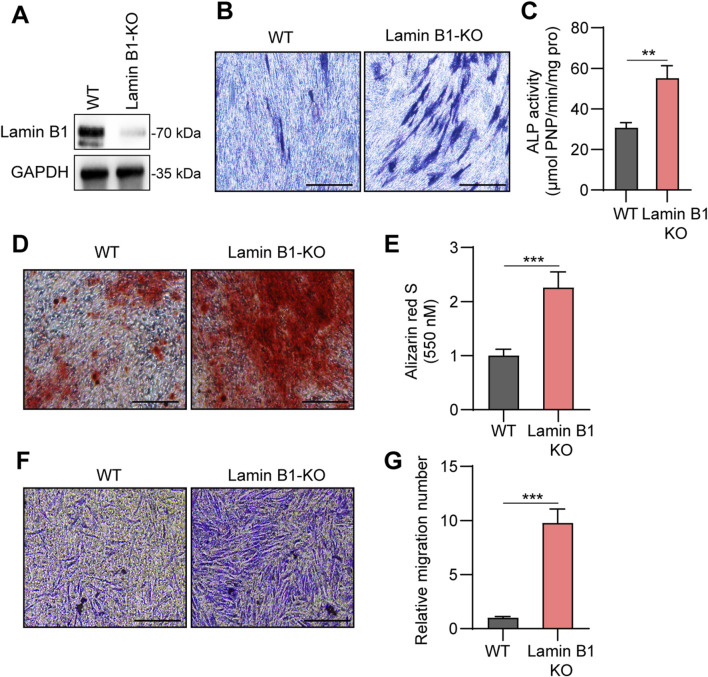
Lamin B1 knockout enhances osteogenic differentiation and migration capacity of primary MSCs. **(A)** Western blot analysis confirming Lamin B1 knockout in primary MSCs (Lamin B1-KO) compared with wild-type (WT) controls, with GAPDH as a loading control. **(B)** Alkaline phosphatase (ALP) staining in WT and Lamin B1-KO MSCs after 7 days of osteogenic induction. **(C)** Quantitative analysis of alkaline phosphatase (ALP) activity in WT and Lamin B1-KO MSCs. **(D,E)** Alizarin Red S staining **(D)** and corresponding quantification **(E)** of mineral deposition in WT and Lamin B1-KO MSCs. **(F,G)** Transwell migration images and quantification in WT and Lamin B1-KO MSCs. Data are presented as means ± SD, *P < 0.05, **P < 0.01 and ***P < 0.001.

To directly determine the functional consequences of Lamin B1 loss, we generated Lamin B1 knockout (Lamin B1-KO) primary MSCs. Western blotting confirmed complete deletion of Lamin B1 in KO cells (Figure 3A). Lamin B1-KO MSCs exhibited significantly enhanced osteogenic potential. Alkaline phosphatase (ALP) activity, an early osteogenic indicator, was markedly higher in KO MSCs (Figures 3B,C). Following 21 days of osteogenic induction, KO MSCs formed more abundant and intense Alizarin Red–positive mineralized nodules compared with wild-type (WT) MSCs (Figure 3D). Quantification of mineralized matrix deposition confirmed significantly elevated mineralization in the KO group (Figure 3E). In addition to enhanced differentiation, Lamin B1 deletion improved MSC migratory capacity. Transwell assay demonstrated substantially greater migration of KO MSCs through the Transwell membrane than WT MSCs (Figures 3F,G). These results demonstrate that Lamin B1 acts as a suppressor of MSC osteogenesis and migration, and its depletion significantly enhances both functional attributes.

### Lamin B1 interacted with KAT2A and decreased its protein stability through ubiquitin-dependent regulation

**FIGURE 4:**
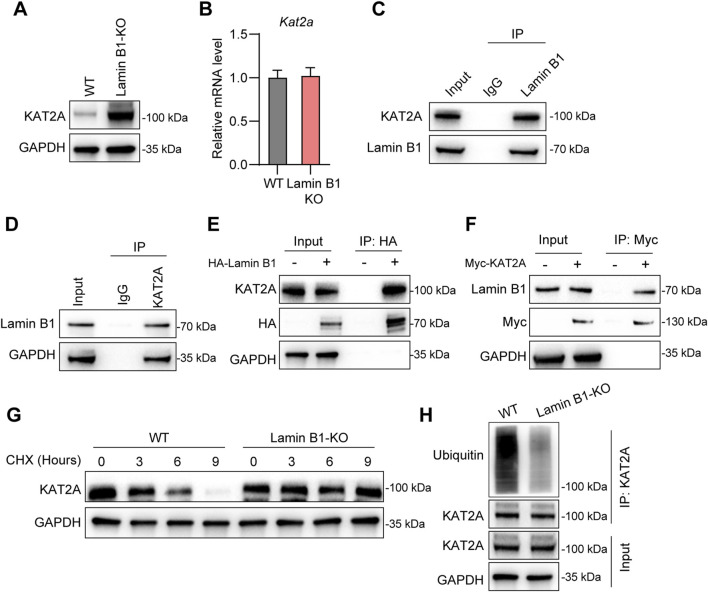
Lamin B1 regulates the interaction with KAT2A and modulates its protein stability. **(A)** Western blot analysis of KAT2A protein expression in wild-type (WT) and Lamin B1 knockout (KO) cells, with GAPDH as a loading control. **(B)** Quantitative analysis of *Kat2a* mRNA levels in WT and Lamin B1-KO cells. **(C)** Co-immunoprecipitation (IP) assay showing the interaction between endogenous KAT2A and Lamin B1 in WT cells, with IgG as a negative control. **(D)** Co-immunoprecipitation assay examining the association between Lamin B1 and KAT2A in WT cells, with IgG as a negative control. **(E)** Co-immunoprecipitation assay using HA-tagged Lamin B1 and Myc-tagged KAT2A in transfected cells to assess their protein–protein interaction, with GAPDH as a loading control. **(F)** Co-immunoprecipitation assay evaluating the interaction between endogenous Lamin B1 and Myc-tagged KAT2A in transfected cells, with GAPDH as a loading control. **(G)** Cycloheximide (CHX) chase assay assessing the effect of Lamin B1 knockout on KAT2A protein stability across different time points following CHX treatment. **(H)** Ubiquitination assay examining the levels of KAT2A polyubiquitination in WT and Lamin B1-KO cells, with GAPDH as a loading control.

To elucidate the mechanism by which Lamin B1 suppresses MSC function, we investigated its potential interaction with the histone acetyltransferase KAT2A, a known regulator of osteogenic gene expression. Lamin B1-KO MSCs exhibited markedly elevated KAT2A protein levels compared with WT controls, despite unchanged *Kat2a* mRNA expression (Figures 4A,B). This discrepancy suggested post-transcriptional regulation. Co-immunoprecipitation assays confirmed that endogenous Lamin B1 physically interacts with KAT2A in MSCs (Figures 4C,D). This interaction was further validated using HA-tagged Lamin B1 and Myc-tagged KAT2A in transfected cells, where both proteins robustly co-precipitated (Figures 4E,F). Cycloheximide (CHX) chase experiments demonstrated that KAT2A protein was significantly more stable in Lamin B1-KO MSCs, suggesting that Lamin B1 promotes KAT2A protein degradation (Figure 4G). Consistently, ubiquitination assays showed markedly reduced poly-ubiquitination of KAT2A in KO cells compared with WT controls (Figure 4H), confirming that Lamin B1 facilitates the ubiquitin-dependent turnover of KAT2A. These findings establish that Lamin B1 negatively regulates MSC osteogenesis by interacting with KAT2A and promoting its ubiquitin-mediated degradation, thereby reducing KAT2A protein stability.

### Transcriptome profiling reveals Lamin B1 as a key regulator of osteogenic and migration-related gene networks

**FIGURE 5:**
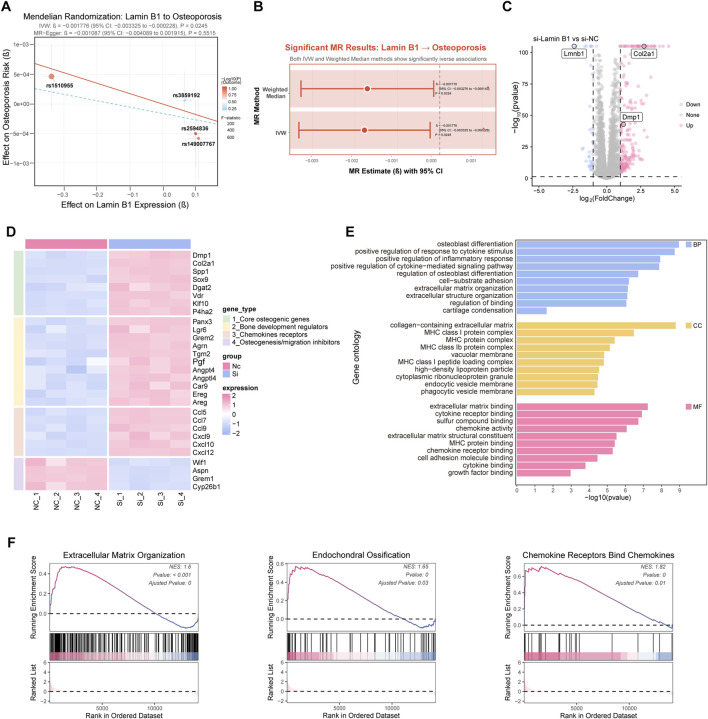
Lamin B1 expression is genetically associated with osteoporosis and regulates gene programs related to osteogenic differentiation and migration. **(A)** Mendelian Randomization analysis evaluating the association between Lamin B1 expression and osteoporosis risk. **(B)** Summary of significant Mendelian Randomization results assessing the causal effect of Lamin B1 on osteoporosis using IVW and Weighted Median methods. **(C)** Differential gene expression analysis in osteoblasts following siRNA-mediated Lamin B1 knockdown (si-Lamb1) compared with control siRNA (si-NC), shown as a volcano plot highlighting upregulated and downregulated genes. **(D)** Heatmap depicting differentially expressed genes between si-Lamb1 and si-NC groups, organized by functional categories including osteogenic regulators, bone development genes, chemokine receptors, and inhibitors of osteogenesis or migration. **(E)** Gene Ontology (GO) enrichment analysis of differentially expressed genes, including biological process (BP), cellular component (CC), and molecular function (MF) categories relevant to osteoblast differentiation, extracellular matrix organization, and cell migration. **(F)** Running enrichment score (RES) plots for selected gene sets, including extracellular matrix organization, endochondral ossification, and chemokine receptor pathways, illustrating pathway-level enrichment patterns.

To further characterize Lamin B1-dependent gene expression changes, we conducted transcriptomic analyses following siRNA-mediated Lamin B1 knockdown. Mendelian randomization analysis first revealed a significant inverse association between Lamin B1 expression and osteoporosis risk, suggesting a functional contribution of Lamin B1 to bone loss conditions (Figures 5A,B). Transcriptomic profiling identified a large panel of differentially expressed genes (DEGs) upon Lamin B1 suppression (Figure 5C). Heatmap visualization revealed that multiple osteogenic regulators and chemokine receptor genes were upregulated, whereas inhibitors of osteogenesis and migration were downregulated (Figure 5D). GO enrichment analysis showed significant overrepresentation of biological processes associated with osteoblast differentiation, extracellular matrix organization, cell migration, and skeletal development (Figure 5E). Gene set enrichment analysis (GSEA) further demonstrated strong enrichment of gene sets related to extracellular matrix organization, endochondral ossification, and chemokine receptor signaling (Figure 5F). Together, these transcriptomic findings indicate that Lamin B1 represses osteogenic and migration-associated transcriptional networks, and its loss shifts MSC gene expression toward a pro-osteogenic, pro-migration state.

### BMSCs with Lamin B1 depletion promoted bone regeneration in an OVX-induced osteoporotic mouse model

**FIGURE 6:**
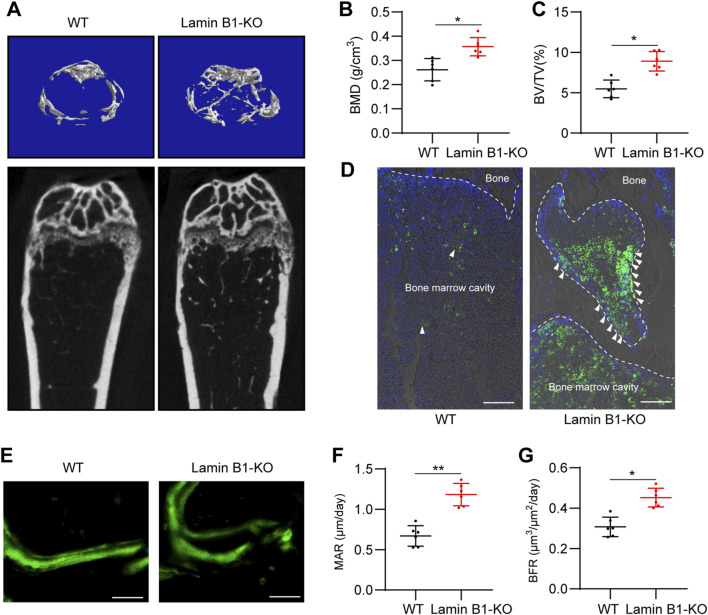
Lamin B1 knockout (KO) enhances bone mass and bone formation in OVX mice. **(A)** Representative micro-CT reconstructions and sagittal sections of the proximal femur from wild-type (WT) and Lamin B1-KO OVX mice 12 weeks after surgery. **(B,C)** Quantification of femoral trabecular bone parameters, including bone mineral density (BMD) **(B)** and bone volume fraction (BV/TV) **(C)** (n = 6). **(D)** Representative immunofluorescence images of femoral sections stained for GFP-labeled donor cells (green) and DAPI nuclear counterstaining (blue). Lamin B1-KO donor cells show more extensive engraftment and linear arrangement along bone surfaces (arrowheads), whereas WT donor cells remain sparsely distributed. **(E)** Representative calcein fluorescent labeling of newly formed bone in WT and Lamin B1-KO mice. **(F,G)** Dynamic histomorphometry analyses showing increased mineral apposition rate (MAR) **(F)** and bone formation rate (BFR) **(G)** in Lamin B1-KO mice. Data are presented as mean ± SD. *P < 0.05, **P < 0.01.

To determine whether Lamin B1 deficiency enhances the therapeutic efficacy of MSCs *in vivo*, we performed intra–bone marrow transplantation of GFP-labeled wild-type (WT) or Lamin B1-knockout (KO) primary MSCs into the femoral cavity of OVX mice. Four weeks after transplantation, micro-CT analysis revealed marked improvements in femoral trabecular architecture in mice receiving Lamin B1-KO MSCs compared with those receiving WT MSCs. Three-dimensional reconstructions and sagittal micro-CT sections showed visibly denser trabecular and improved structural integrity in the KO-MSCs–treated group (Figure 6A). Quantitative assessment confirmed significantly higher bone mineral density (BMD) and bone volume fraction (BV/TV) in the KO-MSC group, indicating an enhanced capacity of Lamin B1-deficient MSCs to restore bone mass in the osteoporotic environment (Figures 6B,C).

To evaluate donor cell engraftment, immunofluorescence staining for GFP was performed on decalcified femoral sections. Lamin B1-KO MSCs exhibited markedly improved engraftment efficiency, accumulating in greater numbers along bone surfaces and forming linear arrangements characteristic of osteogenic niche integration. In contrast, WT MSCs were more sparsely distributed and showed limited alignment with remodeling surfaces, suggesting inferior homing or retention capacity (Figure 6D). These observations indicate that Lamin B1 deletion enhances not only the osteogenic potential of MSCs but also their capacity for bone-surface colonization and microenvironmental interaction *in vivo*.

Dynamic histomorphometry further supported the enhanced regenerative activity of Lamin B1-KO MSCs. Calcein double labeling revealed wider inter-label distances in KO-MSC–treated mice, consistent with increased mineral deposition (Figure 6E). Quantitative analysis confirmed a significantly higher mineral apposition rate (MAR) and bone formation rate (BFR) in the KO group compared with WT MSC recipients (Figures 6F,G). Together, these findings demonstrate that Lamin B1 deficiency substantially improves both the engraftment capacity and bone-forming activity of transplanted MSCs, resulting in effective restoration of bone mass and enhancement of bone turnover in osteoporotic mice.

## Discussion

In this study we identify Lamin B1 as an inhibitory regulator of osteogenic differentiation and bone regenerative function in MSCs. Lamin B1 deficiency enhanced alkaline phosphatase activity, mineral deposition, and MSC migration, and it improved bone formation in an ovariectomy-induced osteoporotic mouse model. These findings indicate that Lamin B1 acts as a molecular brake on MSC osteogenesis and suggest that modulating nuclear lamina composition may strengthen the regenerative potential of MSC-based therapies.

Although nuclear lamins have traditionally been regarded as structural components of the nucleus, recent studies demonstrate that they also influence lineage specification. Prior work has focused primarily on Lamin A/C, which promotes osteogenic differentiation and restricts adipogenesis by regulating nuclear stiffness and transcription factor accessibility (Akter et al., 2009; Swift et al., 2013; Khan et al., 2021). By comparison, the role of B-type lamins has remained less defined. Our data show that Lamin B1 suppresses osteogenic commitment, a function consistent with its role in maintaining nuclear rigidity and stabilizing peripheral heterochromatin. Reduced Lamin B1 may permit chromatin reorganization and enhance accessibility to osteogenic transcriptional programs, aligning with observations that more deformable nuclei support increased lineage plasticity (Harada et al., 2014; Heo et al., 2016).

Beyond mechanical effects, Lamin B1 likely influences MSC differentiation through its impact on chromatin architecture (Pascual-Reguant et al., 2018). Lamin B1 anchors heterochromatin to the nuclear periphery through lamina-associated domains, thereby limiting transcriptional activity (Guelen et al., 2008; Peric-Hupkes et al., 2010; Kind et al., 2013). Its depletion could release osteogenic genes from repressive domains and create a transcriptionally permissive environment. The observed activation of osteogenic and mechanotransduction pathways in our transcriptomic analysis supports this model. Although direct measurements of chromatin accessibility were not performed, these data suggest that Lamin B1 regulates MSC fate by integrating nuclear mechanics with epigenetic control.

Interestingly, we observed an apparent contrast between the physiological downregulation of Lamin B1 during osteogenic differentiation and its pathological upregulation in OVX-derived MSCs. This discrepancy may reflect context-dependent regulation of nuclear architecture. In a healthy differentiation setting, Lamin B1 reduction may facilitate chromatin remodeling and lineage commitment. In contrast, under osteoporotic or stress conditions, elevated Lamin B1 could represent maladaptive nuclear stiffening or stress-associated chromatin stabilization, thereby restricting MSC plasticity and impairing regenerative capacity. Such pathological upregulation may contribute to stem cell dysfunction in osteoporosis and further supports the therapeutic rationale for modulating Lamin B1 activity.

The *in vivo* OVX experiments further demonstrate the translational relevance of Lamin B1 modulation. Lamin B1–deficient MSCs substantially improved trabecular bone structure, bone mineral density, and dynamic histomorphometric indicators of bone formation, outperforming wild-type MSCs. These improvements occurred without exogenous stimulatory factors, indicating that Lamin B1 depletion intrinsically enhances both the engraftment and osteogenic activity of MSCs in an osteoporotic environment. Given the current limitations of MSC-based therapies, targeting Lamin B1 may offer a practical means to increase therapeutic potency.

Despite these strengths, several limitations remain. The downstream transcriptional networks activated by Lamin B1 loss are not fully characterized, and long-term safety concerns—such as genomic instability, senescence induction, or unintended lineage shifts—require further study. Moreover, whether transient Lamin B1 inhibition, rather than permanent gene editing, can achieve similar therapeutic benefits remains unknown. The specificity of Lamin B1 effects on osteogenesis versus other MSC lineages also warrants investigation, as changes in adipogenic or chondrogenic potential may influence overall bone health, particularly in metabolic bone disease.

In the future, small-molecule modulators or RNA-based approaches that transiently suppress Lamin B1 expression may provide a safer and clinically adaptable alternative to permanent gene editing strategies. In conclusion, our results provide mechanistic insight into how nuclear lamina components regulate MSC fate and bone regeneration. They also establish a foundation for developing Lamin B1–targeted strategies to enhance MSC therapy for osteoporosis and related skeletal disorders.

## Methods

### Animals and OVX models

8 weeks female C57BL/6J mice were accommodated within the Laboratory Animal House at the Southern University of Science and Technology, which maintained a controlled temperature environment and a 12-hour light/dark cycle. Food and water were available *ad libitum*. Bilateral ovariectomy (OVX) was performed on female C57BL/6J mice at 8 weeks of age to establish a postmenopausal osteoporosis model. OVX was performed under anesthetic, we performed two dorsolateral incisions to locate and exteriorize two ovaries in OVX group, and sham group underwent sham surgery. Upon removal of the ovaries, the peritoneal cavity and skin were closed. Mice were allowed to recover and were maintained for 8 weeks post-surgery to allow the development of osteoporotic phenotypes. All mice were euthanized at 16 weeks of age for subsequent analyses. All *in vivo* experimental protocols received approval from the Institutional Animal Care and Use Committee (IACUC) of the Southern University of Science and Technology.

### MSCs isolation and culture

Bone marrow–derived mesenchymal stem cells (MSCs) were isolated from female C57BL/6J mice at 8 weeks of age. Briefly, femurs and tibiae were harvested under sterile conditions, bone marrow cells were flushed out using α-MEM supplemented with 10% FBS and 1% penicillin–streptomycin. After 24 h incubation, nonadherent cells were removed and adherent cells were replenished with complete growth medium composed of α-MEM, 10% FBS (Corning), and 1% penicillin/streptomycin (pen/strep, Hyclone). Passages 1 and 2 were used in the following experiments (Fu et al., 2018).

### Intra–bone marrow transplantation of MSCs

For intra–bone marrow transplantation, recipient OVX mice at 16 weeks of age were anesthetized with and placed in a supine position. After sterilization of the surgical site, a small incision was made over the proximal tibia. A 29-gauge needle was used to carefully penetrate the tibial plateau to access the bone marrow cavity. Lamin B1 knockout MSCs or control MSCs were resuspended in sterile PBS, and 4 × 10^5^ cells were slowly injected into the tibial marrow cavity using a Hamilton syringe. Mice were monitored daily after transplantation. Bone samples were collected after 4 weeks post-transplantation for micro-CT and histological analyses.

### Micro-CT analysis

Mouse femurs were immersed fixed in 4% paraformaldehyde (PFA). Micro-CT analyses were performed using a high-resolution µCT scanner (Bruker, Germany). Images were captured at a 10 μm resolution under settings of 60 kV/100 mA through a 0.5 mm aluminum filter. Scanned images were reconstructed by NRecon software, and a three-dimensional model was constructed by CTvox software with the same thresholds for each sample.

### Inducing osteogenic differentiation and ALP detection

To induce osteogenic differentiation, MSCs were incubated in an induction medium comprised of α-MEM, 10% FBS, 10 mM β-glycerophosphate, 50 μg/mL ascorbic acid, and 100 nM dexamethasone (Sigma-Aldrich). ALP staining was performed with 5-bromo-4-chloro-3-indolyl phosphate/nitroblue tetrazolium (Beyotime, Jiangsu, China). For ALP activity, cells were lysed by RIPA lysis buffer (Beyotime) and incubated with p-nitrophenylphosphate (pNPP, Beyotime) substrate. Cellular ALP activity was normalized against total protein concentration measured using a BCA protein assay kit (Pierce, MA, USA).

### Alizarin red S staining

MSCs were cultured with osteogenesis induction medium for 21 days and then were fixed with 10% (v/v) formaldehyde and stained with 2% Alizarin red S (Sigma-Aldrich) at pH 4.2 to evaluate cell matrix calcium mineralization. The culture plates were photographed by an inverted microscope and camera system (Nikon, JP).

### RNA extraction and quantitative RT-PCR analysis

Total RNA was extracted from cultured cells using Trizol reagents (TransGen, CN). Synthesis of cDNA was performed using 2 μg of RNA by a Transcriptor First Strand cDNA Synthesis Kit (Takara, JP) according to the manufacturer’s instructions. Relative mRNA expression levels were determined by applying a SYBR Green qPCR kit (Transgen, CN) using β-actin as reference. Sequences were determined with the CFX96 Real-Time System (Bio-Rad, CA, USA).

### Generation of Lamin B1 knockout MSCs

Lamin B1 knockout MSCs were generated using CRISPR/Cas9-mediated genome editing. sgRNAs (CAT​CGA​TAA​GGT​GCG​CAG​CC) targeting mouse *Lmnb1* were cloned into a lentiCRISPRv2 backbone. Lentiviral particles were produced in HEK293T cells using psPAX2 and pMD2.G packaging plasmids. MSCs were transduced and selected with puromycin (2 μg/mL) for 5–7 days. GFP was introduced for cell-tracking in transplantation experiments.

### Western blot

Western blot was performed as previously described (Fu et al., 2020). Briefly, total proteins were separated by SDS-PAGE gel and transferred onto a polyvinylidene fluoride (PVDF) membrane (Millipore, USA) using a Trans-Blot Turbo^TM^ (Bio-Rad, USA). Subsequently, the PVDF membrane was blocked with a solution of 5% non-fat dry milk in TBS-T for 1 h at room temperature. After blocking, the membrane was incubated overnight at 4 °C with primary antibodies. The membrane was washed three times with TBS-T, followed by incubation with the appropriate HRP-conjugated secondary antibodies for 1 h at room temperature. The blots were developed using an enhanced chemiluminescence ECL kit (ABclonal, China) and imaged with a chemiluminescence imaging system (Tanon, CN).

### Co-IP assay

Co-IP assay was performed as previously described (Huang et al., 2025). Briefly, cells after different treatments were lysed using IP lysis buffer (Thermo Fisher Scientific, USA) supplemented with a proteinase inhibitor cocktail (NCE, China). After centrifugation, the supernatant was incubated with the corresponding primary antibody overnight at 4 °C and then coupled to Protein A/G Magnetic Beads (Thermo Fisher Scientific, USA) at room temperature for 1 h. To reduce nonspecific binding, the beads were extensively washed five times using a DynaMag™-2 Magnet (Thermo Fisher Scientific, USA). The immunoprecipitated complexes were then resuspended in a loading buffer, heated at 95 °C for 5 min, and subsequently analyzed by SDS-PAGE and Western blotting.

### Transwell assay

Cells (5 × 10^3^ cells/well) were added to the top chamber of 24-well Transwell plates with 8 μm pore polyester membrane (Jet, CN). α-MEM with 20% FBS was added to the bottom chamber to induce migration. Cells that had passed through the membrane were stained with 0.1% crystal violet (Aladdin, CN) after being fixed with 4% paraformaldehyde (Beyotime, CN). Images were acquired using an inverted light microscope (Nikon, JP).

### RNA-seq analysis

Total RNA was extracted from cells using TRIzol reagent (Invitrogen, USA) according to the manufacturer’s instructions. RNA quality and integrity were assessed using a Nanodrop 2000 spectrophotometer (Thermo Fisher Scientific, USA) and an Agilent 2100 Bioanalyzer, and only samples with an RNA Integrity Number (RIN) ≥8.0 were used for library preparation. RNA-seq libraries were constructed with the NEBNext Ultra II RNA Library Prep Kit (New England Biolabs) and sequenced on the Illumina NovaSeq 6000 platform to generate 150 bp paired-end reads. Raw reads were evaluated with FastQC (v0.11.9), and adapters and low-quality bases were removed using Trimmomatic. Clean reads were aligned to the mouse reference genome (GRCm38/mm10) using HISAT2, and gene expression levels were quantified with featureCounts from the Subread package to obtain raw read counts. Differential expression analysis was performed using DESeq2 (v1.30.0) in R (v4.1.0), with normalization by the median ratio method. Genes with an average base expression >1 were retained, and the Wald test was applied to identify differentially expressed genes (DEGs), defined as those with |log_2_ fold change| >1 and P < 0.05. Gene annotation, including conversion of gene IDs to symbols and retrieval of gene descriptions, was conducted using the org.Mm.eg.db package (v3.12.0). Gene Ontology (GO) and GSEA analyses were performed using clusterProfiler and MSigDB gene sets.

### Statistical analysis

All numerical data are presented as the mean ± SD. Two groups comparisons were performed using a two-sided unpaired t-test. Multiple comparisons were assessed using one-way ANOVA with Tukey’s *post hoc* test. Statistical significance was defined as *P* < 0.05. All experiments were repeated at least three times unless otherwise specified.

## Data Availability

The original contributions presented in the study are publicly available. This data can be found at the National Genomics Data Center (https://ngdc.cncb.ac.cn/bioproject) under accession number PRJCA060742.
